# Adenocarcinoma of the Right Colon in a Patient with Bloom Syndrome

**DOI:** 10.1155/2016/3176842

**Published:** 2016-08-15

**Authors:** Carlos Augusto Real Martinez, Lilian Vital Pinheiro, Debora Helena Rossi, Michel Gardere Camargo, Maria de Lourdes Setsuko Ayrizono, Raquel Franco Leal, Cláudio Saddy Rodrigues Coy

**Affiliations:** ^1^Division of Colorectal Surgery, University of Campinas, Campinas, SP, Brazil; ^2^Postgraduate Program in Health Sciences, São Francisco University, Avenida São Francisco de Assis, 218 Jardim São José, Bragança Paulista, 12916-350 São Paulo, SP, Brazil

## Abstract

*Introduction*. Bloom syndrome (BS) is an inherited disorder due to mutation in* BLM* gene. The diagnosis of BS should be considered in patients with growth retardation of prenatal onset, a photosensitive rash in a butterfly distribution over the cheeks, and an increased risk of cancer at an early age. Clinical manifestations also include short stature, dolichocephaly, prominent ears, micrognathia, malar hypoplasia and a high-pitched voice, immunodeficiency, type II diabetes, and hypogonadism associated with male infertility and female subfertility. The aim of this report is to describe case of patient with BS who developed adenocarcinoma of the cecum, successfully treated by right colectomy.* Case Report*. A 40-year-old man underwent colonoscopy to investigate the cause of his diarrhea, weight loss, and anemia. The patient knew that he was a carrier of BS diagnosed at young age. The colonoscopy showed an expansive and vegetating mass with 5.5 cm in diameter, located within the ascending colon. Histopathological analysis of tissue fragments collected during colonoscopy confirmed the presence of tubular adenocarcinoma, and he was referred for an oncological right colectomy. The procedure was performed without complications, and the patient was discharged on the fifth postoperative day. Histopathological examination of the surgical specimen confirmed the presence of a grade II tubular adenocarcinoma (stage IIA). The patient is currently well five years after surgery, without clinical or endoscopic signs of relapse in a multidisciplinary approach for the monitoring of comorbidities related to BS.* Conclusion*. Despite the development of colorectal cancer to be, a possibility rarely described the present case shows the need for early screening for colorectal cancer in all patients affected by BS.

## 1. Introduction

Bloom syndrome (BS) is an extremely rare autosomal recessive genetic disorder caused by a mutation in the* BLM* gene encoding a DNA repair enzyme homology to the RecQ helicases that receives the same name of the gene [1, 2]. The absence of BLM protein activity leads to a DNA repair defect, which causes increased risk of mutations [2, 3]. The main clinical features in the BS include short stature, dolichocephaly (long, narrow head), sun-sensitive malar telangiectatic erythema, and immune deficiency, low birth weight, prenatal and postnatal retardation of the growth development, prominent ears, micrognathia, congenital telangiectatic erythema in face, type II diabetes, male infertility and female subfertility, immunodeficiency, upper respiratory recurrent infections, and an increased risk of cancer at an early age [4, 5].

One of the reasons for the interest in this rare syndrome is the great risk to development of malignancies at various sites, most commonly breast, gastrointestinal tract, and skin [6]. Patients with BS are estimated to develop malignancy at a rate 150–300 times higher than the general population and about 25% of them develop malignancy, at a mean age of 20.7 years [6, 7]. This increased risk of cancer leads to a shortened life expectancy and patients with BS rarely survived after their fifties [2]. Approximately 12% of BS patients can develop colon cancer, and the mean age at diagnosis is 35.4 years [2, 8]. The aim of this report is to present a patient with a BS who developed colon cancer at 40 years of age.

## 2. Case Report

**Figure 1:**
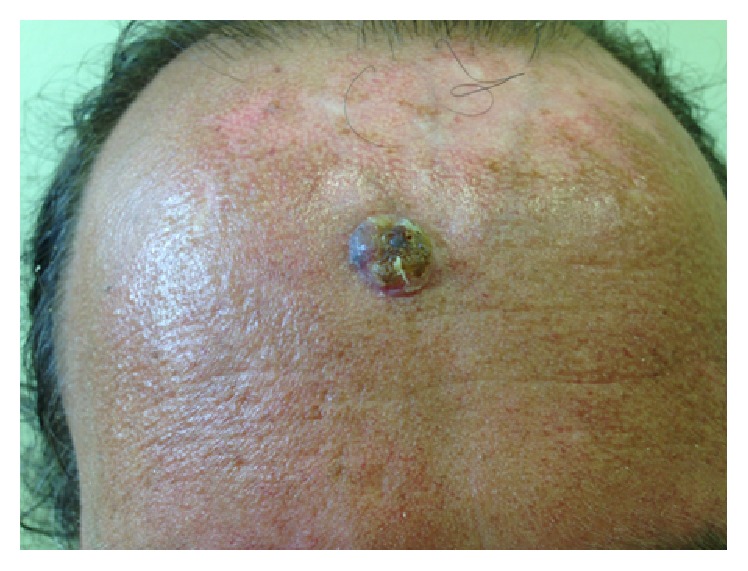
Basal cell carcinoma in forehead.

A 34-year-old-man, with a previous history of recurrent chest infections, presented with a six-month history of diarrhea, abdominal pain specially located in the right lower quadrant, asthenia, lack of appetite, and weight loss (5 kg). He was the second child of four children and referred that one of his brother died at the age of 40 years by liver failure devoid hepatic metastasis of colorectal cancer. He reports type II diabetes making use of metformin. During the adolescence he had been hospitalized on four occasions for the treatment of pulmonary infection. The patient reports that he had recently been referred for surgical resection of a skin tumor with 1.5 cm in diameter located on the forehead (Figure 1). The lesion was removed a week after its diagnosis and the histopathological study confirmed the presence of a basal cell carcinoma. He was born in Brazil and their ancestors do not belong to Ashkenazi ethnicity.

**Figure 2:**
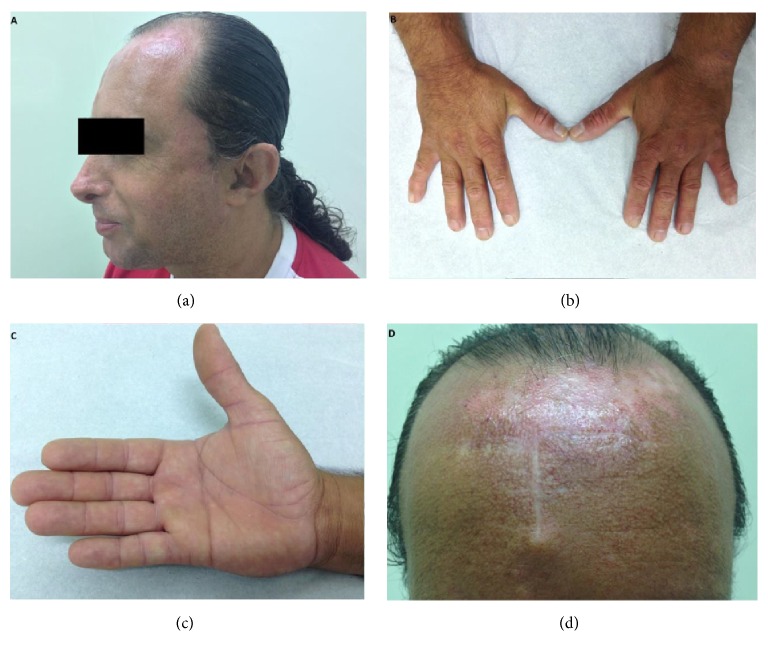
(a) Dolichocephaly, beaked nose, and prominent ears. (b) Clinodactyly and dystrophic nails. (c) Palmar transverse crease. (d) Telangiectatic erythema and linear scar in forehead due to previous surgical excision of basal cell carcinoma.

**Figure 3:**
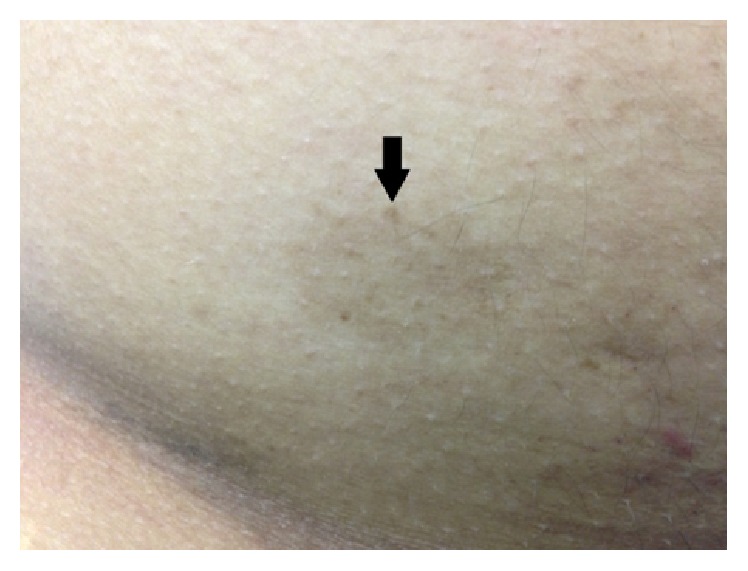
*Café au lait* macules in the anterior abdominal skin.

At admission, physical examination showed a short stature (142 cm), weight of 48 kg, growth retardation, dolichocephaly, triangular face, beaked nose, prominent ears (Figure 2(a)), and hypogonadism. He had a high-pitched voice, small mandible, dystrophic nails, clinodactyly (Figure 2(b)), and palmar transverse crease (Figure 2(c)). He had a telangiectatic erythema in malar region of the face and forehead that worsened after solar radiation and a linear scar in brow related to previous surgical resection of a basal cell carcinoma (Figure 2(d)). He also presents with several* café au lait* macules in the abdominal skin and gluteal region (Figure 3).

**Figure 4:**
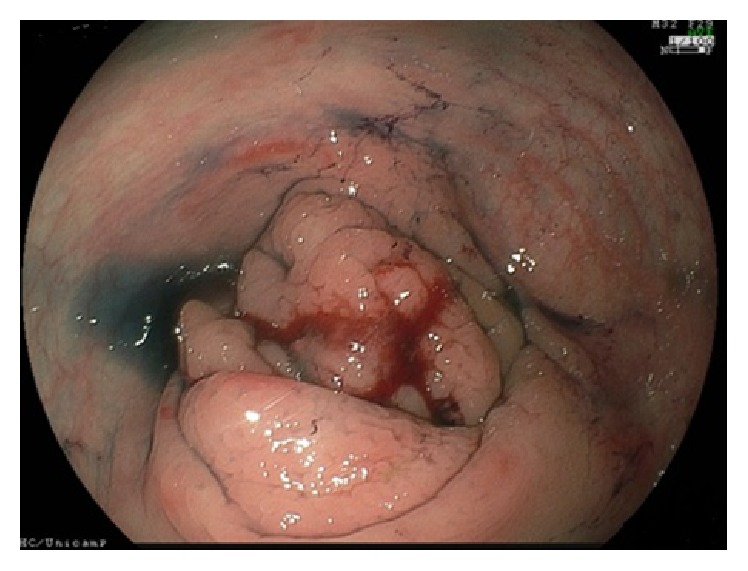
Polypoid-ulcerated mass in ascending colon.

Abdominal examination revealed palpable mobile mass in the right lower quadrant. Blood tests revealed hypoalbuminemia at 1.8 g/L, glycemia at 280 mg/dL, and hypochromic microcytic anemia at 8.4 g/dL. Renal and hepatic biochemical tests were normal. The suspect of BS was confirmed by chromosomic analysis showing chromosomal breakages and sister chromatid exchanges. Upper gastrointestinal endoscopy was normal and colonoscopy revealed a polypoid-ulcerated mass located in ascending colon (Figure 4). Biopsies specimens of the mass confirmed a well-differentiated adenocarcinoma. Computed tomography scan of the abdomen and pelvis only showed the cecal mass without hepatic metastasis or regional lymph node enlargement.

With a diagnosis of ascending colon adenocarcinoma he was submitted to an open oncological right colectomy. He had uneventful recovery and was discharged on the 5th day. The histopathological examination confirmed the diagnosis of tubular adenocarcinoma without neoplastic involvement in 23 lymph nodes resected (stage IIA). The revision of histopathology slide of the tumor located on forehead confirmed the previous diagnosis of basal cell carcinoma. The patient was instructed to daily use sunscreen on all skin surfaces exposed to solar radiation. He was referred to surveillance program of the digestive tract neoplasms, performing colonoscopy and upper endoscopy every year. At this time the patient is well, with no signs of recurrence of the colon cancer or polyps five years after colectomy.

## 3. Discussion

BS also known as congenital telangiectatic erythema is a rare, autosomal recessive genetic disorder found in humans and experimental models [6, 9, 10]. The disease was first described in New York City, in 1954, by David Bloom, a Polish-born dermatologist, as a congenital telangiectatic erythema resembling lupus erythematosus in dwarfs [9, 11].

BS is caused by mutations in* BLM* gene located in chromosome 15 (15q26.1) that comprises 4,437 base pairs, which encodes a protein BLM with 1,417 amino acids homology to the RecQ helicases [12]. Thisprotein restores breaks in double-stranded DNA and is the only known RecQL helicase that can unwind asingle-stranded DNA sequence of four consecutive guanines [13]. The absence of BLM activity leads to a DNA defect repair, which causes genomic instability with increased rates of chromosomal breakage, rearrangements, gene mutation, and increase of the risk of cancer development [3]. The most characteristic cytogenetic features in BS is the 15-fold increased rate of sister chromatid exchanges and this increased level is a pathognomic feature of BS [7, 14]. The transmission of BS is autosomal recessive, and a homozygous state is fundamental for clinical manifestation 1. Heterozygous carriers usually are asymptomatic; however a link between heterozygous carrier status and increased risk of colorectal and breast cancer has been established [1, 15, 16].

The syndrome was initially reported in the Ashkenazi Jewish population. The* BLM *gene mutation is known to be highly prevalent among Ashkenazi population, where the carrier state is estimated to be more than 1 : 110 to 1 : 231 [1, 17–19]. The BS population has been monitored since 1960 via Bloom's Syndrome Registry (BSR) and the data is periodically updated [20]. Actually data collected in 2009 from the BSR showed 265 persons with BS are from 222 families (sibships) [11]. This data also showed that of the 265 cases collected of the BS only 21 (7.9%) patients live in South America and 16 (6.03%) in Brazil [11]. The patient of this report did not belong to the Judaic ethnicity.

The hallmark of the clinical presentation of BS is characteristic with marked retardation of the growth development and an early onset photosensitive facial rash that worsened with solar exposition [1]. Data from BSR evaluating the height in 95 patients showed that the means in men was 149 cm (128–164) while in women was 138 cm (115–160) [11]. The patient described in this report had height of 142 cm, staying within the described mean of the BSR data. The malar telangiectatic erythema of patients with BS usually more intensely affects skin exposed to solar irradiation. Similarly to that found in patients with lupus, the erythema of the patients with BS compromises more frequently the face, especially malar region. The erythema may also compromise the skin of the forehead, as occurs in the patient of this report, ears, lips, and neck [7, 9, 16]. These injuries worsened after sun exposure that can form painful superficial skin ulcers. To prevent the worsening of the telangiectatic erythema and to avoid the increased risk of basal cell carcinoma, as occurs in the patient of this report BS patients need to daily use sunscreen. German reviewing the first 100 cases of BS found eight cases of skin cases and only three cases associated with colorectal cancer [6]. Therefore, the simultaneity of colorectal cancer and skin cancer is a not common association. Other skin lesions are described in patients with BS. The presence of* café au lait* macules mainly located in the trunk is described as found in the patient of this report [7]. Old scars can also be identified at the sites where prevailing telangiectatic erythema resulted from healing of sun exposure ulcers.

Patients with BS generally present with a small but proportionate body, associated dolichocephaly, kneel-shaped face, sharp nose, and low-set ears giving those affected characteristic facies a high-pitched voice, features also on the patient in this case [1, 6]. Type II diabetes mellitus and recurrent respiratory infections are described generally at young age [6, 17]. The patient of this report was referred treatment for type II diabetes since age of 23 years and four previous hospital admissions to treat pneumonia. Repetitive lung infections are related to immunodeficiency often found in patients with BS. Men with BS present with genital hypoplasia and oligospermia. There are reports of adult female patients with BS with a history of normal childbirth, but, generally, the subfertility is a rule [21].

The most significant implication of a diagnosis of BS from the patient's point of view is the high risk of developing cancer [1, 6]. Patients with BS have an increased risk of developing a variety of malignancies, notably leukemia, lymphomas, and carcinomas of various sites, that is high from the twenties onwards, which is the case of our patient. The course of life in these patients is made remarkable by early onset single or multiple neoplasias involving any organ system, which is the main cause of mortality. Many patients will have more than one cancer and most will die by the third decade of life [6, 22]. Newer data indicate that BLM mutations contribute to breast cancer susceptibility, and heterozygous carriers of a BLM mutation have a higher probability of developing colorectal cancer [15, 16, 23]. Among the most serious complications that patients with BS develop lifelong, malignant neoplasms of different locations are the most frequent, accounting for 46%, followed by type II diabetes (15.8%) and lung infections (2.6%) [11]. BSR data showed that of 265 patients followed 122 (46.3%) developed cancer [11]. Of those patients 69 (56.5%) were men and 53 (43.4%) women. Epithelial tumors are the most frequent type (52.5%), followed by lymphoid (24.9%) and hematopoietic neoplasm (11.3%) [11]. Different types of neoplasms are described in about 12% of patients. Acute leukemia and lymphomas seem to predominate in the first two decades of life and carcinomas, mainly located on gastrointestinal tract, more common after the second decade [24]. Tumors that are rare in the general population, such osteosarcoma, medulloblastoma, and Wilms' tumor are more common in patients with BS [9].

About 12% of BS patients develop colorectal cancer, usually from the second decade of life and the median age at diagnosis is 35.4 years, similar to that occurring in patient of this report [8, 24]. German in 1997 analyzing the first 100 cases of cancer on the BSR found 13 cases of colorectal cancer, seven in proximal colon (cecum, ascending, hepatic flexure, and transverse) and six in distal colon (descending, sigmoid, and rectum) [6]. Since then, we can only find three other published cases, two with fatal course [2, 8, 9]. Although the occurrence of skin cancer in patients with BS is well documented in the worldwide literature, the finding of primary skin carcinoma occurring concomitant with colorectal cancer, to the best of our knowledge, only two cases, has been reported [2, 6].

The best surgical approach of colorectal cancer in carriers BS is still controversial. As these patients have an increased risk for developing a second malignance at young age, some authors recommend conducting a proctocolectomy [2]. If the patient refuses the total colectomy partial colectomy and annual follow-up colonoscopy for colon and rectum remaining can be offered. In patient of this report, we discuss the advantages and disadvantages of the two surgical options, and the patient opted for the realization of the right colectomy with annual follow-up. So far, this strategy seemed to us valid because the annual colonoscopic and upper endoscopy monitoring conducted in the last five years did not find any suspicious lesion. This is the first BS case associated with colon and basal cell skin cancer that has been reported in Brazilian population. It is important to recognize this rare condition in order to diagnose the disease at an early stage and tailor the treatment regimen to try to avoid the development of malignances. A screening program should therefore be offered, particularly for upper gastrointestinal, breast, and colon cancer.
